# Plant Sterols as Anticancer Nutrients: Evidence for Their Role in Breast Cancer

**DOI:** 10.3390/nu5020359

**Published:** 2013-01-31

**Authors:** Bruce J. Grattan

**Affiliations:** Department of Family Medicine, Stony Brook University Hospital Medical Center, Stony Brook, New York, NY 11794, USA; E-Mail: bruce.grattan@stonybrookmedicine.edu; Tel.: +1-631-444-8245; Fax: +1-631-444-7552

**Keywords:** plant sterols, cholesterol, cancer, breast cancer, beta-sitosterol, AMPK, bisphosphonates, statins

## Abstract

While many factors are involved in the etiology of cancer, it has been clearly established that diet significantly impacts one’s risk for this disease. More recently, specific food components have been identified which are uniquely beneficial in mitigating the risk of specific cancer subtypes. Plant sterols are well known for their effects on blood cholesterol levels, however research into their potential role in mitigating cancer risk remains in its infancy. As outlined in this review, the cholesterol modulating actions of plant sterols may overlap with their anti-cancer actions. Breast cancer is the most common malignancy affecting women and there remains a need for effective adjuvant therapies for this disease, for which plant sterols may play a distinctive role.

## 1. Introduction

While many factors are involved in the etiology of cancer, it has been clearly established that diet significantly impacts risk of this disease [1,2,3]. The age-adjusted incidence of cancer in the US is 3 times higher than that in Asian countries, with immigrants to the US having increased risk for this condition [4,5]. This suggests critical roles for dietary and lifestyle factors. Despite the results of recent studies failing to demonstrate a large inverse association between produce consumption and overall cancer risk [6], the benefits of key nutrition components unique to plant foods, may still prove beneficial in reducing individual risk and may mitigate the risk of specific cancer subtypes.

There is compelling evidence that increased produce consumption may be associated with a reduction in breast cancer risk [7,8,9]. Specific food components such as sulfurophane [10], indole-3-carbinol [11], and lignans [12] have been investigated in both epidemiological and *in vitro* studies with regards to their effects on breast cancer. Studies such as by Torres-Sanchez have found an inverse correlation between consumption of phytochemicals and risk of breast cancer among postmenopausal women [13] suggesting a potential role for plant sterols in mitigating risk. Plant sterols may be one such nutritional component which may likewise play a role in attenuating breast cancer risk or perhaps serve a chemotherapeutic role.

## 2. Plant Sterols

Plant sterols (PS) are C28 and C29 carbon steroid alcohols [14] that are integral components of plant cell membranes, have been shown to be key components of plant plasma membrane microdomains [15], and may exert similar functions in human cells. These compounds cannot be synthesized by humans and are introduced through the diet where they are found concentrated in plant foods, especially those with are lipid rich [16]. In the American diet, vegetable oils and nuts are particularly significant contributors to plant sterol intake [17,18]. While a variety of PS exist, campesterol, stigmasterol and β-sitosterol are the most abundant PS in the diet, with the prevalence of β-sitosterol being particularly noteworthy [19]. Globally, dietary intake of these compounds has been estimated to be between 200 and 400 mg daily [20], making their intake similar in quantity to cholesterol. PS exist in both the sterol and stanol forms, with the bioavailability of sterols [21] and their dietary prevalence exceeding that of plant stanols [22].

The absorption of PS has been estimated to be 0.4%–3.5% [23] whereas the absorption of phytostanols ranges between 0.02% and 0.3% [24]. In general, the absorption of dietary sterols of plant or animal origin varies significantly. While between 45% and 55% of dietary cholesterol is absorbed, less than 20% and 7% of campesterol and β-sitosterol, respectively, are absorbed [25]. Among healthy subjects, supplementation with 2–3 g of PS has been shown to elevate serum levels of sitosterol and campesterol by 30% and 70%, respectively [26,27]. This limited absorption of PS is the result of their subsequent efflux from enterocytes, a process mediated by ATP binding cassette transports, ABCG5 and ABCG8 [28,29,30] resulting in plasma PS levels of <1 mg/dL [31]. Despite the relatively low circulating quantities of these compounds, they are still capable of exerting important biological effects. The typical American diet has been estimated to provide approximately 80 mg of PS daily, a value significantly lower than that of the Japanese (400 mg) or that seen among vegetarians (345 mg) [32], whose dietary patterns are consistent with a reduction in their breast cancer risk [33].

## 3. Plant Sterols and Cancer

Recent meta-analyses have concluded that doses of plant sterol or stanols of 1–2 g daily can effectively lower LDL-cholesterol levels 8%–12% [34,35,36]. However, despite the relatively strong evidence for a beneficial effect of these compounds on cardiovascular disease risk, these compounds have received comparably little attention with regards to their potential role in cancer etiology. Notwithstanding, the studies to date of PS role in cancer have been promising. The increasing evidence of the biochemical and molecular effects of PS may make them strong candidates for cancer therapy.

With regards to the effects of PS on prostate cancer epidemiological data has failed to find a correlation despite promising *in vitro* data [37,38]. In animal models of colon cancer, PS have been shown to exert beneficial effects [39]. In a case-control study by Mendilaharsu a 50% reduction (95% CI 0.31–0.70) in the risk of lung cancer was seen among those with the highest quartile intake of phytosterols [40]. Similarly, case-control work by De Stefani [41] assessing the effects of PS on stomach cancer risk showed an odds ratio of 0.33 (95% CI 0.17–0.65) among those with the highest PS intake. Populations such as Seventh Day Adventists, who have a lower cancer risk that that of the general population, have likewise been found to have greater intakes of PS [42]. While limited, epidemiological data suggests a correlation between plant sterol intake and a reduction in cancer risk. It has been estimated that phytochemical intake may be related to a reduction in cancer risk upwards of 20% [43]. Despite the promising evidence for PS in other cancer subtypes, the effects of these compounds on the etiology of breast cancer is less well known.

## 4. Plant Sterols and Breast Cancer

Breast cancer is the most common malignancy affecting women, with an incidence of approximately 1/4 of all cancer cases and 14% of all cancer deaths [44]. There are approximately 1 million newly diagnosed cases of breast cancer annually [45]. Even among patients diagnosed with node negative breast cancer, the 10 year estimated risk of recurrence ranges upwards of 50 percent [46]. Indicating a role of environmental exposure and nutritional status among other potential contributors, breast cancer incidence among women in Western countries is 6-fold greater than among women in Asia [47,48]. The dietary contribution of PS may be one nutritional factor affecting the distribution of this disease.

## 5. Estrogen and Breast Cancer

Estrogen is a significant and well recognized mediator of breast cell growth [49,50,51] and as such this hormone plays an important role in the etiology of breast cancer with elevated levels being recognized as a potentially modifiable risk factor for breast cancer [2,52]. A prophylactic effect tamoxifen treatment in those at high risk for developing the disease has been demonstrated in large clinical trials [53]. In those with mutations in the BRCA1 and BRCA2 genes, who are therefore at high risk, prophylactic oophorectomy has been shown to help mitigate risk [54]. At the cellular level, estrogen and estrogen metabolites are directly carcinogenic to breast cells [55,56]. However, recent epidemiological data from the Women’s Health Initiative (WHI) has called into question some of the previous thinking with regards to the effects of estrogen therapy on breast cancer risk suggesting that estrogen monotherapy and the timing of its administration may be important mediators of estrogen’s risk for breast cancer [57]. Notwithstanding, while the WHI trial concluded that estrogen alone was associated with a decreased risk of breast cancer, there are numerous methodological concerns with these data [58,59,60,61,62].

There are two divergent isoforms of the estrogen receptors (ER); alpha and beta, and the receptor to which estradiol binds is an important determinant of this hormone’s cellular effects. ER-α and β are the receptors for estrogen and function as ligand-dependent transcription factors, with the β receptor being considered inhibitory in its effects on breast cell growth [40,63] and the expression of the α receptor being suggested by some to be inversely correlated with breast cancer risk [64]. Epidemiological evidence for this is supported in a 2011 study by Goss *et al.* [65] which demonstrated a significant reduction in the risk of breast cancer in postmenopausal women at increased risk for this disease when treated with the aromatase inhibitor exemestane. Opposing effects are observed between ER-α and the ER-β form, with the alpha form of the ER being the predominant form involved in the proliferative effects of estrogen on cancer growth [66]. Induction of pathways through ER-β on the contrary induces apoptosis/growth arrest and partly underlies the chemopreventative effects of phytoestrogens [67,68].

### Plant Sterols and Estrogen

A number of studies have shown that dietary components can influence ER status [69,70,71,72]. The ER-α is necessary for the proliferative effects of estradiol in breast cancer cells and is overexpressed in the transformed state [73,74,75,76].

It has been shown that intake of β-sitosterol is associated with a greater likelihood of estrogen receptor positive (ER+) than estrogen receptor negative (ER−) tumors (OR 0.49; 95% CI 0.18–0.98) [77]. From the standpoint that the presence of the ER maintains cell responsiveness to endocrine therapy, such as with the selective ER modulator (SERM), tamoxifen, ER+ breast cancer itself represents a more treatable condition than the ER− phenotype. ER− breast cancer is not susceptible to such treatment [78]. This fact underlies the current rationale for ER+ breast cancer treatments, which are aimed at minimizing the utility of this hormone and its resulting stimulatory effects on cell growth and division. However, despite the causal role of estrogen in the progression of ER+ breast cancer, nearly 30%–40% of breast cancers do not exhibit ER+ status [79]. Therefore, newer treatments and/or adjuvant therapies, which do not solely rely upon the ER, are of great importance. 

β-Sitosterol has been demonstrated to competitively bind with equivalent affinity to both the α and β-isoforms of the ER and with an affinity comparable to that of coumestrol [80], which itself has been found to moderately stimulate growth of the ER+ cell line, MCF-7 [81]. Despite exerting an affinity for the ER, in rat models of PS exposure, β-sitosterol failed to increase uterine weight, a marker of estrogenic activity [82]. Likewise, plant stanols and stanol esters failed to stimulate estrogen responsive growth in MCF-7 cells [83]. There is, however some evidence for the estrogenic effects of PS [84], with evidence from reporter gene array studies in human breast cancer cell lines suggesting a role for PS as weak SERMs [80]. Additionally, PS may affect levels of sterol 27 hydroxylase (CYP27A1), an endogenous SERM [85], as β-sitosterol has been shown to inhibit the activity of sterol 27 hydroxylase upwards of 50% [86]. Despite the potential for PS to exert some estrogenic effects, these compounds may still exert beneficial effects on breast cancer, considering that tamoxifen, for instance is also known to be a SERM [87].

While PS may bind the ER or even act as SERMs, there is also the potential for PS to attenuate *de novo* steroid synthesis through reductions in cholesterol levels. To this end, some evidence exists for a reduction in androgens as a result of statin treatment [88], however direct evidence of PS exerting this modality has, to date, not been demonstrated. As will be discussed, PS may indirectly affect estrogen levels through means other than ER binding.

## 6. Plant Sterols and the Liver X Receptor

PS and stanols have been demonstrated to activate both the α (NR1H3) and β (NR1H2) isoforms of the liver X receptor (LXR) [89], whose classical agonists have been oxysterols and their derivatives. LXR-β is ubiquitously expressed [90] whereas expression of the alpha isoform is tissue specific [91,92]. This may be an additional mechanism by which PS may affect estrogen levels, cell division and breast cancer risk. Accumulating evidence has not only demonstrated the expression of LXR in both ER+ and ER− breast cancer cells but suggests that LXR agonists profoundly inhibit cell proliferation [93,94,95]. Mechanistically, LXR activation has been shown to down-regulate the ER while also increasing protein levels of P53 [93]. LXR activation affects multiple regulators of the cell cycle ultimately leading to arrest at G1 [93,95]. Such activation, which may similarly result from PS administration, also results in augmented hepatic cholesterol catabolism [96,97], which may in turn diminish cell proliferation through limiting the availability of the cholesterol needed for cell membrane production.

In addition, the hepatic LXR has been shown to regulate estrogen sulfotransferase, a mechanism through which LXR agonists (such as PS) may induce estrogen deprivation, as sulfonated estrogen is incapable of binding to the ER and activating gene transcription [98]. Furthermore, in xenograft models of invasive ER+ breast cancer (MCF-7/VEGF), LXR activation with a synthetic agonist resulted in a loss of estrogen induced tumorigenicity [99]. While these effects were not observed in ER-MDA-MB-231 cells, LXR is expressed in both cell lines. This suggests the importance of hepatic LXR in regulating systemic estrogen levels and being of particular importance to ER+ breast cancer. The influence of PS on LXR and subsequent estrogen metabolism is of great clinical important given that medications such as tamoxifen are limited in effectiveness with a relapse recurrence rate of approximately 50 percent [100].

## 7. Plant Sterols, the Immune System, and Inflammation

The immune system plays a vital role in cancer etiology with chronic inflammation being recognized as a fundamental aspect of the disease [101,102,103]. The immune system has a pivotal role in cancer prevention and prognosis [104,105,106] and it has been shown that regulatory T cells are both elevated in cancer patients and negatively associated with survival [107,108]. The Th1 axis has an established role in tumor suppression and in patients with HIV a sterol/sterolin mixture was found to increase secretion of Th1 axis cytokines *in vivo* [109].

Likewise, it is recognized that immune system dysregulation plays an important role in cancer metastasis. Interleukin 2 (IL-2) and interferon-γ (IFN-γ) have been demonstrated in animal models of breast cancer to be important in preventing metastasis [110]. PS have been shown to regulate cytokine secretion leading to increased secretion of both IL-2 and IFN-γ [111,112]. Similarly, liposomal delivery of β-sitosterol in a murine model of melanoma was shown to attenuate metastasis [113]. This occurred despite a lack of phytosterol distribution in the blood. This suggests that PS may stimulate the immune system through improving gut surveillance, as IL-2 levels and NK cell activity were noted to be elevated following liposome administration. Stimulatory effects of PS on cytokine production may thus be a means through which this phytosterol exerts preventive effects on cancer metastasis.

Signal Transducer and Activator of Transcription-3 (STAT3) is often constitutively activated in cancer cells and is a causative agent in the transformation to a cancerous phenotype [114]. The downstream targets of STAT3 signaling are well known to be mediators of cancer initiation and progression such as Cyclin D1/D2, c-Myc, Bcl-xl, and Mcl-1 [115,116]. In multiple myeloma, inhibition of STAT 3 both *in vitro* and *in vivo* has been demonstrated to enhance the expression of pro-apoptotic proteins such as Bax while augmenting sensitivity to chemotherapy induced apoptosis [117]. In breast cancer in particular STATs 1, 3, and 5 are all constitutively activated [118,119]. In response to proinflammatory cytokines, STAT1 is activated leading to the upregulation of the innate immune response [120,121,122]. In addition, such activation is linked with the EGFR [123]. Signaling through the EGFR has become an increasing important target for breast cancer therapy. MAPK and PI3K pathways are both downstream targets of EGFR activation [124], and in relation to its link to breast cancer, over expression of the EGFR in MCF-7 cells directly leads to a cancerous phenotype and one that is estrogen independent [125]. Furthermore, the expression of the EGFR is twice as likely in breast cancers which are “double negative”, lacking both the ER and the progesterone receptor (PR) [126]. Indeed tamoxifen resistant MCF-7 cells exhibit upregulation of the EGFR, supporting the hypothesis that upregulation of the EGFR and its related signaling events offer a means of escaping the limits of estrogen-mediated growth [127]. Both EGFR and Her2 are members of the ErbB family of receptors. In highly aggressive breast cancers, Her2 is constitutively activated [128], where it shares tyrosine kinase activity as a binding partner with the EGFR [129]. In fact, it has been estimated that Her2 is amplified in 25%–30% of all breast cancer cases [130,131]. With regards to its immunologic effects, Her-2 overexpression is known to downregulate the major histocompatability complex class I (MHC-I) thereby reducing immune surveillance of breast cancer cells [132,133]. Plant sterols have been shown to activate AMPK in a manner similar to metformin, and may thus act as metformin mimetics. To this end, metformin has been shown to rescue MHC-I from downregulation by Her-2 overexpression in breast cancer cells [134].

Recently, β-sitosterol has been shown to decrease the nuclear translocation of nuclear factor kappa B (NF-κB) [135] which promotes inflammation, is constitutively activated in this disease and leads to a more aggressive, hormone independent phenotype [136,137]. In addition, downregulation of NF-κB *in vivo* has been shown to increase cancer cell susceptibility to the apoptotic effects of tumor necrosis factor alpha (TNF-α) [138] while also minimizing cell metastatic capability through modulation of growth factors and cytokines. Downregulation of NF-κB inhibits the production of vascular endothelial growth factor, interleukin-8, interleukin-6, and matrix metalloproteinase-9 (MMP9) [139,140] each of which are implicated in breast cancer.

## 8. The Effect of Plant Sterols on Cholesterol: Implications for Cancer

The most well recognized clinical outcome of PS intake is their hypocholesterolemic effects. Indeed in cultured enterocytes, sitosterol has been shown to decrease the expression of the Niemann-Pick C1-Like 1 transporter [141]. However the effects of PS on cholesterol may, in turn, mediate their potential anticancer effects. Awad and colleagues [142], have shown the capability of β-sitosterol to suppress growth and to induce apoptosis in MDA-MB-231 cells, suggesting a potential role for dietary constituents as adjuvant therapy for this disease. Following β-sitosterol treatment, a 66% reduction in cell growth was noted. Upwards of an 87% reduction in breast cancer cell growth was noted in another study by Awad utilizing the estrogen responsive MCF-7 cell line [143]. The fact that these effects persisted despite the differences in estrogen responsiveness between these two cell lines, suggests additional, non-hormonal effects of PS. Similar work by this group demonstrated that β-sitosterol in comparison with campesterol, induced a substantial reduction in the cholesterol fraction of total cellular sterols with β-sitosterol accounting for 75% of total sterols following treatment [144]. This may be an additional mechanism through which PS may affect cell growth and be of utility to breast cancer treatment or prevention.

### 8.1. Cholesterol and Cancer

The effect of PS on cholesterol levels has been recognized since the 1950’s [145] and the effect of PS on cholesterol may, in part, underlie their effects on cancer risk. A historic study by Hinds, found a positive correlation between dietary cholesterol intake and the risk of lung cancer, using a case control design. This finding extended across a variety of ethnic groups and remained even after controlling for age and occupational exposure to lung carcinogens [146]. A recent, large, prospective, study [147] demonstrated a positive correlation between total cholesterol and cancer risk with a hazards ratio of 1.17 being determined for breast cancer in particular (95% CI 1.03–1.33). Others have shown that every 10 mg/dL decrement in LDL is associated with a 15% (95% CI 12%–18%) reduction in cancer risk (*p* < 0.001) [148]. The ability of PS to reduce LDL cholesterol has been shown to occur with an average reduction of 8.8% [149]. To this end, statins, which lower LDL cholesterol an average of 1.8 mmol/L [150], have been suggested to lower cancer risk upwards of 50% [151,152]. The effect of PS is specific to LDL with no effect on HDL levels [153], however, through their LDL effects, PS consumption increases the relative levels of HDL. Consistent with these effects, an inverse relationship has been demonstrated between HDL cholesterol and cancer risk, with every 10 mg/dL increase being associated with a 36 percent reduction in overt risk (95% CI 24%–47%) [148]. Similarly, other studies have corroborated the association between increased HDL and diminished cancer risk [154].

Cholesterol is an integral component of cellular membranes and thus, demand for cholesterol is augmented during periods of rapid cellular proliferation [155]. Among many potential mechanisms, cholesterol has been shown to reduce levels of MMP-1 [156], the serum levels of which are negatively associated with survival among breast cancer patients [157].

Likewise, it has been recognized for some time that depletion of cholesterol has inhibitory effects on cellular growth [158,159,160]. Inhibition of HMG-CoA-reductase by lovastatin has been found to consistently induce G1 arrest to the degree that such treatment has been suggested as an experimental means of cell cycle synchronization [161]. Statins have been suggested to be of use in breast cancer therapy [162,163] and treatment of breast cancer cells with lovastatin led to an overexpression of PTEN and a resulting decrease in AKT/PKB signaling [164]. Cellular cholesterol levels however are tightly regulated. Such regulation occurs through a balance of uptake and synthesis via sensor mechanisms and feedback loops consisting of sterol regulatory element binding protein (SREBP) and SREBP cleavage activating protein (SCAP) [165]. Cholesterol regulates genes necessary for lipid metabolism which contain sterol regulatory elements (SREs) in their promoter regions. Reductions in intracellular cholesterol leads to an activation of endoplasmic reticulum (ER) bound SCAP protein to begin processing of SREBP-1a,-1c,-2. In turn, these factors migrate to the nucleus and influence the transcription of genes through the binding of SRE promoter regions [166,167]. In addition to the statin mediated effect on G1, in human acute promyelocytic leukemia cells (HL-60), and acute lymphoblastic leukemia cells (MOLT-4), there is a specific role for cholesterol in modulating the activity of the p34cdc2 kinase which regulates the G2-M transition [168]. Thus, there are multiple modalities for an effect of cholesterol on cell cycle regulation and overall cellular proliferation for which PS may modulate.

Recently, a number of studies have observed a reduction in breast cancer risk with bisphosphonate use as well as the potential for use of these medications as adjuvant therapies for this disease [169,170]. Likewise, evidence supports a role for bisphosphonates in reducing breast cancer metastasis to bone [171]. Bisphosphonates have a known role in the regulation of cholesterol synthesis. It was first demonstrated by Amin [172] that some bisphosphonates reduce *de novo* cholesterol synthesis through inhibition of squalene synthase, with alendronate and pamidronate being found to inhibit mevalonate as well as squalene synthase. Overlapping with the effects of PS, this work illustrates the role of these medications in the modulation of cellular cholesterol levels. Given the growing evidence for the role of bisphosphonates as a means of breast cancer treatment, or prevention, and the known role of cellular cholesterol metabolism in cell division and growth, the potential exists that these compounds may be exerting their chemotherapeutic effects via mechanisms influencing cellular cholesterol levels. Such a mechanism may be mimicked through intake of PS.

While operating at different points in the mevalonate pathway, treatment with either statins or bisphosphonates leads to a reduction in farnesyl pyrophosphate and geranylgeranyl-pyrphosphate, both of which are required for protein prenylation. Such prenylation has important roles in the generation of lipidated protein domains that enable protein-protein interactions and subsequent cell signaling. The prevention of protein prenylation by either statins or bisphosphonates also leads to endoplasmic reticulum stress as a result of a reduction in prenylated rab proteins [173,174]. Such ER stress results in the initiation of the unfolded protein response [175] and subsequent autophagy [176]. While significant advances have been made in understanding the effects of PS on cholesterol metabolism [177], much remains to be investigated with regards to their effects on breast cancer etiology. While the effects of PS are milder than statins, they may exert similar effects with a far more efficacious safety profile.

### 8.2. Plant Sterols and Oxidative Stress

In addition to their roles in hormone production, cell signaling and cell membrane organization, plant sterols may impart a cellular antioxidant effect. In comparison with noncancerous growths such as fibroadenomas, breast cancer cells have been found to exhibit greater oxidative stress [178]. Furthermore, breast cancer cells have been shown to possess lower levels of coenzyme Q-10, a potent antioxidant, than juxtaposed non cancerous cells, as well as higher malonyldialdehyde levels, indicating increased oxidative damage to lipids [179]. Additionally, breast cancer patients have been found to have significantly lower levels of glutathione and reduced total antioxidant capacity in comparison with healthy controls [180]. However, β-sitosterol has been shown in cell culture studies to modulate levels of both glutathione peroxidase as well as superoxide dismutase (SOD) [181]. Treatment of thymocytes derived from BALB/c mice with β-sitosterol was found to prevent radiation induced nuclear strand tears as well as stimulate antioxidant enzyme systems in these cells including catalase, SOD and glutathione peroxidase, while inhibiting cytochrome c release [182]. β-Sitosterol has also been shown to induce antioxidant defense systems in the pancreas of streptozotocin treated rats [183] and has been shown to protect against the depletion of antioxidants seen in models of chemically induced cancer, while also augmenting tissue levels of nonenzymatic antioxidants [39]. Furthermore, as HMG-CoA reductase inhibitors [184], PS may affect ROS generation in addition to cellular cholesterol content. Both statins [185] and PS [135] have been demonstrated to reduce NF-κB activation and this may be a mechanism underlying the antioxidant effects of PS [186].

## 9. Plant Sterols and Glucose Metabolism

Given the alterations in cell metabolism of some cancers towards glycolytic pathways, as first described by Warburg [187], interventions aimed at modulating glucose signaling, may prove salutary in cancer therapy. Metformin and associated dietary mimetics of this medication, including PS, may be one such therapy.

The biguanide anti-diabetes medication, metformin, has been shown to selectively induce apoptosis among cancer cells [188]. As well, this medication has been shown to reverse the loss of immune system surveillance by way of recovering MHC-I expression [134]. The downregulation of MHC-I is a process that is intertwined with the Warburg effect [189] and thus metformin and metformin mimetics may exert therapeutic effects through both pathways. 

Mechanistically, metformin works in part through activation of adenine monophosphate kinase (AMPK) which is a component of the LKB1/AMPK/mTOR/IRS/Akt pathway [190,191,192,193]. AMPK also increases the AMP/ATP ratio resulting in reduced hepatic glucose output [194], with AMPK being allosterically regulated through binding of AMP to its alpha and gamma subunits [195]. The effects of metformin on the AMP/ATP ratio make this pharmaceutical a mimetic of dietary energy restriction and it has been suggested that metformin may be of clinical utility in breast cancer prevention [196].

It has been observed that plasma levels of β-sitosterol are lower among type 2 diabetics [197], with hypoglycemic effects of β-sitosterol being observed in other studies [198]. β-Sitosterol has been shown to be an AMPK agonist, with the beneficial effects of β-sitosterol on glucose metabolism being mediated, in part, through this mechanism [199]. The agonistic effects of PS on AMPK and the pathways activated by metformin may be another means through which these compounds may exert anticancer effects. Membrane cholesterol content is known to regulate GLUT4, with recent evidence demonstrating that AMPK induced insulin sensitization is in part a result of depletion of membrane cholesterol content [200]. As PS activate AMPK while also displacing membrane cholesterol, PS may have important effects on glucose metabolism and subsequent cell growth. Synthesized variants of PS such as disodium ascorbyl phytostanol phosphate have been shown to reduce body weight as well as cholesterol levels in animal studies [201]. Given the association between energy intake and cancer risk [202,203], such compounds may be of benefit in reducing the risk of both cancer as well as cardiovascular disease. In addition to these mechanisms, AMPK also phosphorylates and inactivates HMG-CoA reductase [204].

In addition to being part of antiapoptotic pathways, AMPK regulates a vital glucose-dependent cell cycle checkpoint at G1/S [205]. The sensitivity of this checkpoint for glucose is such that continued activation of mTOR and amino acid availability are not sufficient for overriding cell arrest at this junction. This finding has important mechanistic implications for PS insomuch as the phosphorylation of P53 by AMPK is required for arrest at this checkpoint and indeed persistent AMPK activation results in cellular senescence [205].

P53 is a well known apoptotic mediator that also plays an important role in stimulating cell cycle arrest induced by DNA damage [206]. Mutations in the phosphoprotein P53 have long been recognized to be prevalent in malignancies. In fact, it is estimated that P53 gene (TP53) mutations occur in nearly 50% of tumors [207]. Furthermore, mutant P53 also has been shown to upregulate the mevalonate pathway [208].

Additionally, through activating AMPK, PS may not only inhibit cell proliferation but enhance cellular antioxidant capacity via FOXO transcription factors such as DAF-16 which is known to possess a sterol sensing domain [209]. In so doing, the subsequent upregulation of catalase, IGFBP1, and MnSOD may limit oxidative damage and stymie aberrant cell growth. Notwithstanding, insulin/IGF signaling, which is upregulated under conditions of insulin resistance, inhibits and subsequently suppresses SKN-1 which is involved in intestinal phase II detoxification [210]. Plant sterols may upregulate FOXO transcription factors through their effects on AMPK [211] and both FOXO1 and FOXO3 have been shown to promote apoptosis during the unfolded protein response (UPR). As discussed, the UPR may be induced following cholesterol depletion, by such means as PS, and FOXOs have been shown to promote apoptosis during endoplasmic reticulum stress through inhibiting the normal increase in NF-κB, which itself exerts anti-apoptotic functions [212].

Insulin resistance is becoming increasingly common and one of the potential mechanisms through which insulin resistance affects cancer risk is through an increase in the bioavailability of IGF-1. Insulin resistance has been linked with an increased incidence of a variety of cancers, including breast cancer [213]. Hyperinsulinemia increases hepatic IGF-1 production while concurrently diminishing IGFBGs [214]. Likewise, insulin reduces sex hormone binding globulin (SHBG) and thus increases the levels of bioavailable estrogen [215]. The inflammatory cytokine profile seen among those who are insulin resistance may also contribute to the transformed state [216]. The AMPK activating effects of PS may improve insulin resistance, thereby reducing IGF-1 levels.

IGFs play an important role in breast cancer etiology having been shown to exert mitogenic, transforming, and antiapoptopic properties, especially when coupled with other growth factors [217]. In addition, it has been demonstrated that overexpression of the IGF-1 receptor (IGF-1R) results in the transformation of non-cancerous cells to ones possessing a malignant phenotype [218]. Further evidence suggest that not only is IGF-1 responsible for induction of MMPs but there is a reciprocal effect between IGF-1 and MMPs such that MMPs function in part to maintain the IGF-1R [218]. Cellular cholesterol depletion, such as by plant sterols, disrupts the antiapoptotic effects of IGF-1 signaling through reducing the levels of phosphoinositol 3 kinase (PI3K) [219].

## 10. Plant Sterols, Membrane Organization, and Cell Signaling

There are several lines of evidence implicating PS in cell membrane organization including sphingolipid and ceramide metabolism and alterations to caveolae. 

### 10.1. Beta Sitosterol and Ceramide Metabolism

Lipids rafts have important effects on cell signaling in breast cancer [220,221] and these moieties are affected by the levels of cellular ceramide. Ceramide is a sphingolipid which is believed to function as a tumor suppressing lipid [222], and has been shown to diminish the cholesterol content of lipid rafts [223]. In response to ceramide availability, PTEN is increased in caveolae enriched microdomains [224] and is known to negatively regulate insulin signaling. Through removing the 39-UTR phosphate of PIP3, PTEN antagonizes PI3K [225]. *In vitro* evidence supports a role for β-sitosterol treatment in inducing a reduction in sphingomyelin via activation of sphingomyelinase, as well as an increase in ceramide [143]. These alterations to the components of lipid rafts have a known role in apoptosis initiation [226,227]. β-Sitosterol has been suggested to operate in part, via modulation of the sphingomyelin cycle, and through alterations in phospholipase A2 [228].

Although ceramide may have antitumor effects, an association has been noted between increased levels of glycoslyated ceramide, glucosylceramide, and resistance to cancer treatment [229,230,231]. Levels of glucosylceramide are regulated by the activity of glucosylceramide synthase and targeted inhibition of this enzyme has been demonstrated to restore cancer cell sensitivity to therapeutics [232,233]. Lucci *et al.* [231] demonstrated elevated levels of glucosylceramide in tumor specimens from patients with breast cancer and melanoma whom were resistant to chemotherapy. Indeed, the increased glycosylation of ceramide may be a mechanism utilized by cancer cells to become drug resistant [234]. Glycosylation of ceramide may provide a means of escaping the growth inhibitory effects of ceramide. Awad demonstrated that combined treatment of tamoxifen with β-sitosterol potentiates the effects of this medication on the growth of MCF-7 cells and MDA-MB-231 cells. β-Sitosterol was found to be a potent activator of serine palmitoyltransferase, the rate limiting enzyme in ceramide synthesis [143]. Additionally, this adjuvant therapy was found to inhibit glucosylceramide synthase, and thus the combination of β-sitosterol and tamoxifen may lower glucosylceramide levels while increasing the relative quantities of nascent ceramide with its subsequent inhibition of breast cancer cell proliferation. Importantly, β-sitosterol was found to be more effective in inhibiting the growth of MDA-MB-231 cells, an ER− cell line with a more aggressive phenotype.

Caveolae are types of lipid rafts which function as platforms for organizing a variety of cell signaling pathways. Caveolae are known to be upregulated in multidrug resistant tumors [235]. The caveolae scaffolding protein Cav-1 contains a 20 amino acid microdomain which enables its binding to signaling molecules [236] and both Cav-1 and P53 have been shown to work in synergy [237]. Caveolin is a marker for caveolae and also functions directly to modulate the actions of a variety of signaling cascades including the ER [238], EGFR [239], src [240] and the insulin receptor (IR) [241]. The effects of IR localization in caveolae and their association with caveolin have been suggested to influence insulin’s downstream mitogenic and metabolic effects [242,243]. Likewise, the IGF-1R has been shown to localize to caveolae [244].

Cholesterol is a major component of caveolae [156]. Lipid raft domains are affected by cholesterol depletion and the signaling moieties associated with them are presumably altered in sequence. The localization and activity of the breast cancer resistance protein (BCRP/ABCG2) has been associated with both cellular cholesterol content as well as proximity to lipid rafts. Similarly, cholesterol depletion been shown to reduce the activity of BCRP by 40% [245]. Changes to the sterol content of caveolae may be a mechanism by which PS may affect signaling pathways involved in both cell metabolism and division

### 10.2. Beta Sitosterol and Apoptosis

β-Sitosterol has been demonstrated to activate Fas. In both MCF-7 and MDA-MB-231 cells, both the expression of Fas protein and the activity of caspase 8, were selectively increased by the addition of β-sitosterol [246]. Fas is a cell surface death receptor whose activation constitutes the extrinsic apoptotic pathway. Fas activation results in the recruitment of intracellular adapter proteins including FADD (Fas Associated Death Domain) and TRADD (TNF receptor associated death domain). Together, these molecular pathways induce caspase-mediated apoptosis [247]. Furthermore, PS may initiate apoptosis through their effects on tumor necrosis factor related apoptosis inducing ligand (TRAIL). TRAIL has been demonstrated to be a potent apoptotic mediator among a variety of cancer phenotypes, while demonstrating minimal effects to normal cells both *in vitro* and *in vivo* [248,249]. TRAIL may exert unique therapeutic effects against breast cancer stem cells [250]. TRAIL mediates its apoptotic effects through caspase 8 activation, with this caspase subsequently inducing a number of effector caspases with protease activity [251,252,253,254]. A study by Awad *et al.* [255] found that β-sitosterol induced apoptotic effect in MDA-MB-231 cells through upregulating caspases 3, 8, and 9. Likewise, β-sitosterol augmented the bax:bcl-2 ratio. Together, low dose β-sitosterol and TRAIL were found to synergistically stimulate apoptosis in MDA-MB-231 cells [256]. In murine xenograft models, β-sitosterol treatment has similarly been found effective in reducing growth of MDA-MB-231 cells [257]. In other studies, the addition of PS to the diet significantly reduced tumor size in several studies in which athymic mice were injected with human breast cancer cells. This process was found to be independent of estrogen signaling [257,258].

## 11. Conclusion

It has been suggested that approximately 35% of cancer deaths are attributable to modifiable risk factors including dietary intake [259] with inconsistencies remaining about the degree to which different nutritional factors and dietary patterns affect this condition. To effectively assess the potential of these compounds, given the potential efficacy of plant sterols as outlined in this review, further research, in particular clinical trials, is warranted.
